# Molecular Subtypes and Treatment Modalities in Breast Cancer in a Brazilian Military Hospital: An Observational Study

**DOI:** 10.1155/bmri/4549789

**Published:** 2026-10-29

**Authors:** Larissa Fernanda Campos Moreira da Silva, Fabiana Pirani Carneiro, Andrea Barretto Motoyama

**Affiliations:** ^1^ Brasília Area Military Hospital (HMAB) Brasília Brazil; ^2^ School of Medicine University of Brasília (UnB) Brasília Brazil unb.br; ^3^ Laboratory of Cancer Molecular Pathology Faculty of Health Sciences Universtiy of Brasilia (UnB) Brasília Brazil; ^4^ Center for Molecular Biotechnology (C-Biotech) Universtiy of Brasilia (UnB) Brasília Brazil

**Keywords:** breast neoplasia, prognosis, molecular subtypes

## Abstract

Breast cancer is a global public health concern and the most common malignancy in women, with a lifetime risk of 12.5%. It is also the most frequently diagnosed cancer, accounting for 11.6% of all cases. This study is aimed at describing the clinical, anatomopathological, and sociodemographic profile of women diagnosed with breast cancer at a public military hospital in Brazil, and evaluating the association between molecular subtypes and the type of treatment received. This was an observational, cross‐sectional, and quantitative study. Data were extracted from the hospital′s electronic medical records (AGHUse platform) in 2022 and 2025, involving a total of 102 women. Most cases were diagnosed at clinical Stage II (49.0%). Regarding molecular classification, the most prevalent subtype was Luminal B (47.1%), followed by Luminal A (18.6%), HER2‐enriched luminal (16.7%), triple negative (12.8%), and HER2‐enriched nonluminal (4.9%). A total of 54.9% of the tumors had histological Grade 2. Most patients (71.6%) did not present metastases, whereas a minority did (28.4%). Treatment decisions were guided by molecular subtypes, with triple negative and HER2‐enriched subtypes more frequently receiving neoadjuvant or palliative treatment and luminal subtypes predominantly treated with adjuvant therapy; no statistically significant association was found between individual molecular subtypes and the overall type of treatment received, which is likely attributable to the limited statistical power resulting from the small sample size within the neoadjuvant subgroup (*n* = 36). The total cohort for neoadjuvant analysis consisted of 36 patients, with a global pathological complete response (pCR) rate of 47.2% (*n* = 17). Within the residual disease group (*n* = 19), qualitative stratification revealed that 73.7% (*n* = 14) achieved a partial response (pPR), whereas 26.3% (*n* = 5) presented a minimal response. Although the global association between molecular subtypes and pathological response (pCR vs. RD) did not reach statistical significance (*p* = 0.2831), a subgroup analysis comparing aggressive phenotypes (triple negative and HER2‐enriched) against luminal phenotypes (Luminal A and B) was evaluated (*p* = 0.169; OR = 3.61; 95% CI: 0.86–15.20). Aggressive subtypes demonstrated a clinically higher rate of pCR (59.1% vs. 28.6%), supporting biological aggressiveness as a driver of chemosensitivity in this cohort. These results contribute to clinical decision‐making and a better understanding of the disease profile in women treated in military hospitals. Moreover, it highlights the need for better response prediction markers for pathological response.

**Synopsis**

This study characterized the clinical, anatomopathological, and sociodemographic profile of 102 women with breast cancer treated at a military hospital in Brazil. Most patients were over 50 years old and in Stage II, with Luminal B being the most frequent molecular subtype. Treatment patterns observed aligned with molecular subtypes (luminals primarily received adjuvant therapy, whereas triple negative/HER2‐enriched were more frequently treated with neoadjuvant or palliative therapy), though a statistically significant association between molecular subtypes and the overall type of treatment was not found in this cohort. This finding underscores the limited statistical power of the small subgroup analysis rather than a true biological equivalence between subtypes. However, when grouped into two categories (“aggressive” vs. “luminal”), a clear clinical trend was observed (59.1% vs. 28.6%; OR = 3.61; *p* = 0.169).

## 1. Introduction

Breast cancer is a global public health issue and the most common malignant neoplasm in women, with a lifetime risk of 12.5%. It accounts for 11.6% of all cancers and is the leading cause of cancer‐related death among women in Western countries [1–3]. Early detection remains a major challenge in improving breast cancer management. Mammography and core needle biopsy are considered gold standards for screening and diagnosis. Despite improvements in screening and treatment, over 50% of patients still experience disease recurrence [4].

Breast cancer encompasses distinct clinical subtypes with morphological, molecular, and biological features that influence prognosis, survival, and clinical outcomes [5]. These characteristics also guide treatment decisions [6, 7].

This study is aimed at investigating the clinical, pathological, and molecular characteristics of women with breast cancer and describing their sociodemographic profiles at a military public hospital. The findings may contribute to public health policies and inform therapeutic strategies for this population.

## 2. Methods

This is an observational, cross‐sectional, retrospective study with a quantitative approach. Variables for data collection were extracted from the institutional database accessed via the Aghuse Information System platform during the period from 2022 to 2025 [8].

The Brasília Area Military Hospital provides care to approximately 21,000 women, primarily soldiers, officers, and employees of the armed forces and their families. From this population, a cohort of 102 women with histologically confirmed breast cancer was identified and included in the present study, allowing for an in‐depth analysis of clinical, histological, and molecular factors related to metastasis and therapeutic management [8]. All variables described herein reflect the patients′ status at the diagnostic time. Because this is a retrospective study, it was possible to follow the disease progression. However, these data are not depicted in the tables and can be provided upon request.

The data were subjected to descriptive analysis and expressed through measures of central tendency and dispersion, along with 95% confidence intervals (CI) and a significance level set at 5%. To evaluate the associations between categorical variables—specifically clinical staging, molecular subtypes, metastatic involvement, and treatment modalities (adjuvant, neoadjuvant, and palliative)—Pearson′s chi‐square test or Fisher′s exact test were employed as appropriate, given the distribution and small frequency counts within specific subgroup cells. Statistical analyses were performed using R software (R Studio), Version 2023.6.1.524. For the global analysis of pathological response across multiple molecular subtypes, the Fisher–Freeman–Halton test was employed. Strength of association was estimated using odds ratios (ORs) with 95% CI.

To evaluate the efficacy of neoadjuvant chemotherapy, pathological response was assessed in surgical specimens. Pathological complete response (pCR) was defined as the complete absence of invasive tumor cells in both breast tissue and lymph nodes (ypT0/is ypN0). For cases presenting persistent disease, the residual tumor burden was qualitatively stratified into two levels based on histopathological reports: partial response (characterized by a substantial reduction in tumor volume but with persistent macroscopic foci or moderate cellularity) and minimal response (characterized by minimal alteration in tumor architecture, maintaining a high residual tumor volume). Categorical variables and response stratifications were analyzed using Fisher′s exact test.

This study received ethical approval for data collection from the Brasília Area Military Hospital and the Research Ethics Committee of the Faculty Health Sciences of the University of Brasília (Approval Number: 7,439,405; CAE: 82582524.7.000.0030).

Understanding the specific molecular subtypes of breast cancer, notably Luminal A and Luminal B, is paramount for prognostic assessment and treatment planning. For each sample, immunohistochemical (IHC) analyses for ER, PgR, HER‐2, and Ki‐67 were performed according to the American College of Pathologists guidelines. To assure a precise distinction between Luminal A and Luminal B types, a Ki‐67 cutoff of 14% was applied to differentiate Luminal A (Ki‐67<14%) from Luminal B (Ki‐67 ≥ 14%). Samples were considered HER2‐positive if they scored 3+ by immunohistochemistry or demonstrated gene amplification via in situ hybridization (ISH/FISH). Furthermore, the Ki‐67 assay itself adhered to internationally standardized protocols, incorporating rigorous quality control procedures—such as the use of positive and negative controls in each batch and meticulous evaluation by experienced pathologists—thereby ensuring the reproducibility and accuracy of the obtained results, critical for robust subtyping [9].

Clinical staging was performed according to the *American Joint Committee on Cancer (AJCC) Staging Manual*, *8th edition*, which integrates anatomic TNM parameters (tumor size, nodal involvement, and distant metastasis) with biological markers (tumor grade, ER, PgR, and HER2 status). For the IHC evaluation, estrogen receptor (ER) and progesterone receptor (PgR) statuses were considered positive if nuclear staining was observed in 1% of tumor cells, following international consensus. HER2 status was defined as positive when scoring 3+ by IHC or presenting gene amplification confirmed by in situ hybridization (ISH/FISH) in cases scored as 2+ (equivocal). Tumors with scores of 0 or 1+ were classified as HER2‐negative [10].

## 3. Results

### 3.1. Clinicopathological Characteristics

The study included 102 patients, with ages ranging from 18 years to > 70 years. The majority were aged 51–70 years (42.16%; *n* = 43), followed by ≤ 50 years (40.19%; *n* = 41) and > 70 years (17.65%; *n* = 18). Regarding clinical staging, 49.02% (*n* = 50) were in Stage II, 29.41% (*n* = 30) in Stage I, 15.69% (*n* = 16) in Stage III, and 5.88% (*n* = 6) in Stage IV.

Metastatic involvement at diagnosis was observed in 28.43% (*n* = 29), whereas 71.57% (*n* = 73) showed no evidence of metastasis. In terms of histological grade, Grade 2 was predominant (54.90%; *n* = 56), followed by Grade 3 (30.39%; *n* = 31) and Grade 1 (12.75%; *n* = 13), with 1.96% (*n* = 2) not evaluated.

According to molecular subtype, the most frequent category was Luminal B (47.06%; *n* = 48), followed by Luminal A (18.63%; *n* = 19), HER2‐enriched luminal (16.67%; *n* = 17), triple negative (12.75%; *n* = 13), and HER2‐enriched nonluminal (4.90%; *n* = 5) (Table 1).

**Table 1:** Clinicopathological characteristics of breast cancer patients. Brasília, Federal District, Brazil, 2025. (*N* = 102).

Characteristic	Category	*n*	%
Age	≤ 50 years	41	40.19%
	51–70 years	43	42.16%
	> 70 years	18	17.65%

Clinical staging	Stage I	30	29.41%
	Stage II	50	49.02%
	Stage III	16	15.69%
	Stage IV	6	5.88%

Metastasis	Metastatic	29	28.43%
	Nonmetastatic	73	71.57%

Histological grade	Grade 1	13	12.75%
	Grade 2	56	54.90%
	Grade 3	31	30.39%
	Not evaluable	2	1.96%

Molecular classification	Luminal A	19	18.63%
	Luminal B	48	47.06%
	HER2‐enriched luminal	17	16.67%
	HER2‐enriched nonluminal	5	4.90%
	Triple negative	13	12.75%

### 3.2. Association Between Clinical Variables and Metastasis

Clinical staging showed a strong correlation with the metastasis at diagnosis. Patients in Stage III (44.83%; *n* = 13) had a significantly higher risk, whereas Stage I was associated with a lower risk. (0%; *n* = 0). Stage II showed a tendency toward reduced risk (34.48%; *n* = 10, OR = 0.43; 95% CI: 0.18–1.06; *p* = 0.064), although without statistical significance (Table 2).

**Table 2:** Association between molecular subtypes staging metastasis and type of treatment. Brasília, Federal District, Brazil, 2025. (*N* = 102).

1. Clinical variable vs. metastasis					
Variable	Category/subtype	Metastatic (*n*)	Nonmetastatic (*n*)	OR (95% CI)	*p*
Clinical staging	I	0	30	0.02 (0.00–0.42)	< 0.001
	II	10	40	0.43 (0.18–1.06)	0.064
	III	13	3	18.96 (4.83–74.5)	< 0.001
	IV	6	0	40.66 (2.20–748)	< 0.001

Molecular subtype	HER2‐enriched luminal	5	12	0.92 (0.26–3.22)	1.000
	HER2‐enriched nonluminal	2	3	1.50 (0.23–9.73)	0.650
	Luminal A	1	18	1.60 (0.18–14.4)	0.990
	Luminal B	13	35	0.88 (0.37–2.09)	0.776
	Triple negative	8	5	5.39 (1.15–25.3)	0.032

Histological grade	Grade 1	0	13	0.07 (0.00–1.29)	0.012
	Grade 2	15	41	Reference	—
	Grade 3	14	17	1.56 (0.65–3.72)	0.320
	Not available	0	2	—	—

Age range	≤ 50 years	13	28	Reference	—
	51–70 years	10	33	0.55 (0.21–1.48)	0.876
	> 70 years	6	12	0.90 (0.26–3.08)	1.000


*Note:* All *p* values calculated using Pearson′s chi‐square or Fisher′s exact test as appropriate. OR not estimable due to zero cell count;*p*value from Fisher′s exact test.

Abbreviations: CI, confidence interval; OR, odds ratio; pCR, pathological complete response.

The triple negative subtype was associated with a fivefold higher risk of metastasis (24.24%; *n* = 8; OR = 5.39), and this association was statistically significant (95% CI: 1.15–25.3; *p* = 0.032). No significant associations were observed for the other subtypes. Regarding histological grade, only Grade 1 showed a protective effect (0%; *n* = 0; OR = 0.07; 95% CI: 0.00–1.29; *p* = 0.012), whereas Grades 2 (51.72%; *n* = 15) and 3 (48.28%; *n* = 14) were not significantly correlated with metastasis (Table 2).

Age was not significantly associated with metastatic disease: Patients aged 51–70 years (34.48%; *n* = 10; OR = 0.55; 95% CI: 0.21–1.48; *p* = 0.876) showed similar risk compared with those ≤ 50 years (44.83%; *n* = 13) and > 70 years (20.69%; *n* = 6; OR = 0.90; 95% CI: 0.26–3.08; *p* = 1.000) (Table 2).

### 3.3. pCR and Metastasis

The total cohort for neoadjuvant therapy analysis consisted of 36 patients, with a global pCR rate of 47.2% (*n* = 17) (Table 3). Detailed analysis of the 19 patients with residual disease revealed that 73.7% (*n* = 14) achieved a partial response (pPR), whereas 26.3% (*n* = 5) had a minimal response. The molecular distribution of the neoadjuvant cohort showed Luminal B (*n* = 13), triple negative (*n* = 12), and HER2‐enriched luminal (*n* = 9) as the primary subtypes. The global association between molecular subtypes and pathological response (pCR vs. RD) was evaluated using the Fisher–Freeman–Halton test, yielding a *p* value of 0.2831, indicating no statistically significant association across all groups. However, a subgroup analysis comparing aggressive phenotypes (triple negative and HER2‐enriched, *n* = 22) against luminal phenotypes (Luminal A and B, *n* = 14) revealed a *p* value of 0.169 (Fisher′s exact test). Aggressive subtypes showed a higher likelihood of achieving pCR (59.1% vs. 28.6%; OR = 3.61; 95% CI: 0.86–15.20) (Table 3).

**Table 3:** Neoadjuvant response (pCR vs. residual disease) according to molecular subtype and subgroup analysis in patients with breast cancer (*n* = 36).

**1. Neoadjuvant response (pCR vs. residual) – updated (n = 36)**					
	**PCR (n)**	**Residual disease**	**pCR rate (%)**	**OR (95% CI)**	**p**
Molecular subtype					
Triple negative	7	5	58.3	2.50 (0.22–28.7)	0.619
HER2‐enriched luminal	5	4	55.5	2.25 (0.18–27.5)	0.680
Luminal B	4	9	30.7	0.80 (0.12–5.20)	0.750
HER2‐enriched nonluminal	1	0	100	—∗	1.000
Luminal A	0	1	0	—∗	1.000
**Overall**	17	19	47.2	1.65 (0.43–6.29)	0.460
**Subgroup analysis**					
**Aggressive (TN + HER2-L)**	13	9	59.1	3.61 (0.86–15.2)	0.169
**Luminal (A + B)**	4	10	28.6	Reference	—
**Overall**	17	19	47.2	Reference	—
**Residual disease stratification (n = 19)**					
**Partial response (pPR)**	—	14	(73.7% of RD)		
**Minimal response**	—	5	(26.3% of RD)		
**2. Type of treatment vs. molecular subtype (n = 102)**					
**Molecular Subtype**	Neoadjuvant (*n*)	Adjuvant (*n*)	Paliative (*n*)		
HER2‐enriched luminal	9	7	1		
HER2‐enriched nonluminal	1	3	1		
Luminal A	1	18	0		
Luminal B	13	32	3		
Triple negative	12	0	1		
Global p value for treatment modality vs. subtype: p = 0.064					

### 3.4. Treatment According To Molecular Subtype

Treatment strategies were tailored according to both clinical staging and molecular subtype. For Luminal A patients, adjuvant therapy was the predominant approach (94.74%; *n* = 18/19), with only a small proportion (5.26%; *n* = 1/19) receiving neoadjuvant treatment. Luminal B patients exhibited a broader distribution of therapies, including adjuvant (66.67%; *n* = 32/48), neoadjuvant (27.08%; *n* = 13/48), and palliative care (6.25%; *n* = 3/48). In the HER2‐enriched luminal group, neoadjuvant therapy (52.94%; *n* = 9/17) was slightly more prevalent than adjuvant (41.18%; *n* = 7/17), whereas palliative treatment was administered to a minority of patients (5.88%; *n* = 1/17). Neoadjuvant therapy was highly more prevalent (92.31%; *n* = 12/13), among the triple negative subtype patients with only one patient (7.69%; *n* = 1/13) receiving palliative care. Finally, for the HER2‐enriched nonluminal subtype patients, adjuvant therapy was the most frequent regimen (60.00%; *n* = 3/5), and neoadjuvant and palliative therapies each accounted for 20.00% (*n* = 1/5) of treatments.

Despite these varying treatment modalities, no statistically significant association was found between the receipt of neoadjuvant therapy and the achievement of pCR across the molecular subtypes (Table 3).

## 4. Discussion

This study describes the sociodemographic, clinical, and therapeutic characteristics of 102 women diagnosed with breast cancer and treated at a public military hospital in the Central‐West Region of Brazil. The military healthcare setting may partially explain the considerable proportion of patients diagnosed before the age of 50 because active personnel and their dependents benefit from regular medical evaluations and facilitated access to healthcare services, potentially enabling earlier diagnosis [11].

Histological Grade 2 was the most frequent grade observed, followed by Grades 3 and 1, consistent with previous reports [12–14]. Histological grade remains an important prognostic factor because it reflects tumor aggressiveness and metastatic potential, particularly when interpreted together with clinical staging.

Regarding molecular classification, Luminal B was the predominant subtype, followed by tumors that were HER2‐enriched luminal, Luminal A, triple‐negative, and HER2‐enriched nonluminal. Similar subtype distributions have been reported in other populations [15–18]. Although Luminal A is generally the most prevalent subtype worldwide [19], Luminal B predominated in our cohort.

Molecular classification at diagnosis serves both as a prognostic factor and for treatment choice [18]. Luminal A subtype, with ER expression and low proliferation index, is a well‐differentiated tumor and, therefore, has better prognosis, better survival rates, and a lower relapse rate among all types [19]. Luminal A is the most prevalent molecular subtype for breast cancer [19]; however, it was not the most frequently found in this study. Luminal B subtype, the most prevalent subtype herein, also expresses ER, but has a high proliferation rate, which confers a worse prognosis than that of Luminal A [20]. Tumors with positive hormone receptors and negative HER2, associated with high proliferation index, have a higher risk of early recurrence, highlighting the need for permanent monitoring and periodic restaging [21]. Triple‐negative and HER2‐enriched subtypes, which usually show far worse prognosis, were found at even lower frequencies in our study, in accordance with the international literature [22].

Therapeutic decisions in breast cancer are based on the integration of disease stage, histological characteristics, molecular subtype, and patient‐related factors [23–25], with gene expression assays further refining adjuvant chemotherapy recommendations [26]. Accordingly, most patients in our cohort received adjuvant treatment, followed by neoadjuvant and palliative approaches. No statistically significant association was observed between molecular subtype and treatment modality. This finding likely reflects both the multifactorial nature of therapeutic decision‐making and the relatively small sample size, which represents an important limitation of this study [23–25, 27].

Regarding the challenges and opportunities of personalized medicine in breast cancer, the literature suggests that treatment choice extends beyond mere molecular characterization of the tumor [22]. Even within a military health organization, where there is easy access to medications, molecular tests, and an institutionalized Brazilian protocol, clinical decisions are, in fact, the result of integrating multiple biological, clinical, and patient‐specific factors, although global disparities in breast cancer care remain a significant challenge [23, 28]. This may explain why molecular subtype alone did not emerge as a statistically significant predictor of the main therapy type (adjuvant, neoadjuvant, or palliative) in our sample, underscoring that the multifactorial nature of the decision persists regardless of access barriers [23].

The persistence of traditional clinicopathological factors as major determinants of treatment selection is noteworthy. Disease stage, nodal status, histological grade, and proliferation index remain fundamental components of treatment planning, even within healthcare systems with broad access to molecular testing and targeted therapies [22, 24]. In this context, neoadjuvant treatment continues to play a critical role by reducing tumor burden, increasing breast‐conservation rates, and allowing assessment of response through pCR [23, 29, 30].

The biological behavior observed among molecular subtypes was consistent with current evidence. Triple‐negative and HER2‐enriched tumors demonstrated more aggressive characteristics and were more frequently selected for neoadjuvant treatment. In contrast, luminal tumors generally exhibited less aggressive features and lower responsiveness to chemotherapy [24, 30–32]. Triple‐negative breast cancer deserves particular attention because it was associated with a higher frequency of metastatic disease in our cohort, reinforcing its recognized adverse prognostic profile [22].

Most patients were diagnosed with nonmetastatic disease, a finding that may also reflect the facilitated access to healthcare services available within the military health system [2, 12, 33]. As expected, metastasis was associated with advanced clinical staging, and our results confirmed the relationship between advanced stage, higher histological grade, and more aggressive disease behavior [10, 22, 34, 35].

Analysis of neoadjuvant therapy revealed patterns consistent with the literature. Although no statistically significant association between molecular subtype and pCR was identified, triple‐negative and HER2‐enriched nonluminal tumors tended to achieve higher complete response rates, whereas Luminal A and Luminal B tumors more frequently exhibited residual disease [29–31], a pattern also observed in comparative analyses of HR+/HER2+ versus HR−/HER2+ tumors [36]. Similar findings have been reported in Latin American cohorts, reinforcing the consistency of these biological patterns across populations [37]. Notably, the subgroup analysis comparing aggressive phenotypes (triple negative and HER2‐enriched) against luminal phenotypes (Luminal A and B) demonstrated a clear clinical trend, though it did not reach statistical significance (*p* = 0.169), with aggressive subtypes showing a higher likelihood of achieving pCR (59.1% vs. 28.6%; OR = 3.61; 95% CI: 0.86–15.20). This finding is consistent with the literature, supporting biological aggressiveness as a driver of chemosensitivity [29–31, 36]. Although the global association across all five molecular subtypes was not statistically significant (*p* = 0.2831), the observed trend from the biologically driven subgroup analysis is consistent with the expected differential response between luminal and aggressive phenotypes. Nonetheless, the small number of patients undergoing neoadjuvant therapy represents a limitation to our study, and larger studies are warranted to confirm these findings.

Overall, our findings reinforce the importance of integrating molecular subtype, histological grade, and clinical stage when assessing prognosis and planning treatment. In particular, the higher metastatic risk observed among triple‐negative tumors and the greater likelihood of pCR among aggressive phenotypes emphasize the value of biological stratification in personalized breast cancer management. Larger studies are warranted to further clarify the factors influencing metastasis and treatment response in this population [34].

## 5. Conclusions

We identified that advanced clinical staging (III and IV) represents significant risk factors for metastasis, whereas histological Grade 1 was associated with a reduced risk in our cohort of patients. The triple‐negative molecular subtype, in particular, was shown to be associated with an increased risk of metastasis, reinforcing the crucial relevance of molecular classification in risk stratification.

The final analysis shows a global pCR rate of 47.2%. Within the residual disease group (*n* = 19), 73.7% achieved a partial response, whereas 26.3% presented a minimal response. The direct comparison between aggressive and luminal phenotypes did not reach statistical significance (*p* = 0.169), although that aggressive subtypes (triple negative and HER2‐enriched) showed a higher likelihood of achieving pCR compared with luminal subtypes in this population (59.1% vs. 28.6%; OR = 3.61; 95% CI: 0.86–15.20). The global analysis across all five molecular subtypes did not reach statistical significance, reflecting the limited statistical power of the small subgroup sample rather than a true biological equivalence between subtypes.

In summary our results underscore the imperative nature of an integrated evaluation of clinical, histological, and molecular characteristics—within the context of available healthcare resources and patient access—to optimize treatment definition and, consequently, the prognosis of breast cancer patients.

## Funding

No funding was received for this manuscript.

## Conflicts of Interest

The authors declare no conflicts of interest.

## Data Availability

The data supporting the findings of this study are already presented within the manuscript. Due to the nature of the study and the need to ensure patient confidentiality, no additional datasets that could allow patient identification can be shared. Any questions, additional information, or specific requests regarding the data may be directed to the corresponding author of the study.

## References

[1] Coughlin S. S. , Social Determinants of Breast Cancer Risk, Stage, and Survival, Breast. (2019) 177, no. 3, 537–548, 10.1007/s10549-019-05340-7. 31270761

[2] Bray F. , Ferlay J. , Soerjomataram I. , Siegel R. L. , Torre L. A. , and Jemal A. , Global Cancer Statistics 2018: GLOBOCAN Estimates of Incidence and Mortality Worldwide for 36 Cancers in 185 Countries, CA: A Cancer Journal for Clinicians. (2018) 68, no. 6, 394–424, 10.3322/caac.21492, 30207593. 30207593

[3] Noone A. M. , Howlader N. , Krapcho M. , Miller D. , Brest A. , Yu M. , Ruhl J. , Tatalovich Z. , Mariotto A. , Lewis D. R. , Chen H. S. , Feuer E. J. , and Cronin K. A. , SEER Cancer Statistics Review (CSR) 1975-2015, 2018, National Cancer Institute.

[4] Uyisenga J. P. , Debit A. , Poulet C. , Frères P. , Poncin A. , Thiry J. , Mutesa L. , Jerusalem G. , Bours V. , and Josse C. , Differences in Plasma MicroRNA Content Impair MicroRNA-Based Signature for Breast Cancer Diagnosis in Cohorts Recruited From Heterogeneous Environmental Sites, Scientific Reports. (2021) 11, no. 1, 10.1038/s41598-021-91278-0, 34083680. PMC8175697 34083680

[5] Chen Y. , Wu N. , Liu L. , Dong H. , and Wu C. , Correlation Between MicroRNA-21, MicroRNA-206 and Estrogen Receptor, Progesterone Receptor, Human Epidermal Growth Factor Receptor 2 in Breast Cancer, Clinical Biochemistry. (2019) 71, 52–57, 10.1016/j.clinbiochem.2019.07.002, 31276668. 31276668

[6] Bozkurt S. B. , Ozturk B. , Kocak N. , and Unlu A. , Differences of Time-Dependent MicroRNA Expressions in Breast Cancer Cells, Non-coding RNA Research. (2021) 6, no. 1, 15–22, 10.1016/j.ncrna.2020.12.001, 33385103. PMC7770513 33385103

[7] Cardoso M. R. , Santos J. C. , Ribeiro M. L. , Talarico M. C. R. , Viana L. R. , and Derchain S. F. M. , A Metabolomic Approach to Predict Breast Cancer Behavior and Chemotherapy Response, International Journal of Molecular Sciences. (2018) 19, no. 2, 10.3390/ijms19020617, 29466297. PMC5855839 29466297

[8] Ministério da Saúde. Diretoria de Saúde: A Saúde da Nossa Força, https://www.dsau.eb.mil.br/.

[9] Maranta A. F. , Broder S. , Fritzsche C. , Knauer M. , Thürlimann B. , Jochum W. , and Ruhstaller T. , Do YOU Know the Ki-67 Index of Your Breast Cancer Patients? Knowledge of Your Institution′s Ki-67 Index Distribution and Its Robustness Is Essential for Decision-Making in Early Breast Cancer, Breast. (2020) 51, 120–126, 10.1016/j.breast.2020.03.005, 32302928. PMC7375657 32302928

[10] Amin M. B. , Edge S. B. , Compton C. C. , Gershenwald J. E. , Brookland R. K. , Meyer L. , Gress D. M. , Byrd D. R. , and Winchester D. P. , The Eighth Edition AJCC Cancer Staging Manual: Continuing To Build A Bridge From A Population‐Based To A More “Personalized” Approach To Cancer Staging, CA-A Cancer Journal for Clinicians. (2017) 67, no. 2, 93–99, 10.3322/caac.21388. 28094848

[11] DeSantis C. E. , Ma J. , Gaudet M. M. , Newman L. A. , Miller K. D. , Goding Sauer A. , Jemal A. , and Siegel R. L. , Breast Cancer Statistics, 2019, CA-A Cancer Journal for Clinicians. (2019) 69, no. 6, 438–451, 10.3322/caac.21583. 31577379

[12] Moreira C. N. , Teixeira M. L. S. , Domingues E. M. , da Silva C. M. F. , Durães A. T. S. , dos Santos V. T. , Souto B. L. C. , and Oliveira D. L. T. Q. , Perfil Clínico-Epidemiológico e Histológico do Câncer de Mama no Estado da Bahia, no Periodo Entre 2011 e 2021, Brazilian Journal of Health Review. (2023) 6, no. 2, 4711–4725, 10.34119/bjhrv6n2-022.

[13] Lages L. P. , Araújo E. R. , Lemos M. H. , Araújo S. G. , Aleluia R. G. , Chaves L. M. , Ribeiro N. , Batista K. , Siqueira A. , and Araujo M. , Perfil dos Índividuos com Câncer de Mama em Idade Jovem no Piauí, Brazilian Journal of Surgery and Clinical Research. (2018) 24, no. 1, 2317–4404.

[14] Abreu E. and Koifman S. , Fatores Prognósticos no Câncer da Mama Feminina, Revista Brasileira de Cancerologia. (2019) 48, no. 1, 113–131, 10.32635/2176-9745.RBC.2002v48n1.2270.

[15] Akoz G. , Diniz G. , Ekmekci S. , Ekin Z. Y. , and Uncel M. , Evaluation of Human Epididymal Secretory Protein 4 Expression According to the Molecular Subtypes (Luminal A, Luminal B, Human Epidermal Growth Factor Receptor 2-Positive, Triple-Negative) of Breast Cancer, Indian Journal of Pathology and Microbiology. (2018) 61, no. 3, 323–329, 10.4103/IJPM.IJPM_465_17, 30004048. 30004048

[16] Al-Hussaini M. , Al-Badran A. , Al-Thiyab H. , Al-Aboudi A. , Al-Mohanna M. , Al-Omran M. , Al-Badran H. , and Al-Eid E. , Prevalence of Breast Cancer Subtypes in the Middle East and North Africa: A Systematic Review and Meta-Analysis, Cancer Research and Treatment. (2022) 54, no. 4, 1125–1135, 10.4143/crt.2022.007.

[17] Hashmi A. A. , Aijaz S. , Khan S. M. , Mahboob R. , Irfan M. , Zafar N. I. , Nisar M. , Siddiqui M. , Edhi M. M. , Faridi N. , and Khan A. , Prognostic Parameters of Luminal A and Luminal B Intrinsic Breast Cancer Subtypes of Pakistani Patients, World Journal of Surgical Oncology. (2018) 16, no. 1, 10.1186/s12957-017-1299-9, 29291744. PMC5749004 29291744

[18] Lundgren C. , Bendahl P. O. , Borg Å. , Ehinger A. , Hegardt C. , Larsson C. , Loman N. , Malmberg M. , Olofsson H. , Saal L. H. , Sjöblom T. , Lindman H. , Klintman M. , Häkkinen J. , Vallon-Christersson J. , Fernö M. , Rydén L. , and Ekholm M. , Agreement Between Molecular Subtyping and Surrogate Subtype Classification: A Contemporary Population-Based Study of ER-Positive/HER2-Negative Primary Breast Cancer, Breast Cancer Research and Treatment. (2019) 178, no. 2, 459–467, 10.1007/s10549-019-05378-7, 31432367. PMC6797629 31432367

[19] Neves Rebello Alves L. , Dummer Meira D. , Poppe Merigueti L. , Correia Casotti M. , do Prado Ventorim D. , Ferreira Figueiredo Almeida J. , Pereira de Sousa V. , Cindra Sant’Ana M. , Gonçalves Coutinho da Cruz R. , Santos Louro L. , Mendonça Santana G. , Erik Santos Louro T. , Evangelista Salazar R. , Ribeiro Campos da Silva D. , Stefani Siqueira Zetum A. , Silva dos Reis Trabach R. , Imbroisi Valle Errera F. , de Paula F. , de Vargas Wolfgramm dos Santos E. , Fagundes de Carvalho E. , and Drumond Louro I. , Biomarkers in Breast Cancer: An Old Story With a New End, Genes. (2023) 14, no. 7, 10.3390/genes14071364, 37510269. PMC10378988 37510269

[20] Rosa K. G. , Teodoro R. D. F. , Marques M. M. , Penna U. P. A. , and Libera L. S. D. , A Importância da Classificação Molecular no Prognóstico do Câncer de Mama: Perspectivas Atuais, Revista Eletrônica da Faculdade de Ceres. (2021) 10, no. 1, 46–70, 10.37951/refacer.v10i1.5879.

[21] Orrantia-Borunda E. , Anchondo-Nuñez P. , Acuña-Aguilar L. E. , Gómez-Valles F. O. , and Ramírez-Valdespino C. A. , Mayrovitz H. N. , Subtypes of Breast Cancer, Breast Cancer, 2022, Exon Publications, 10.36255/exon-publications-breast-cancer-subtypes. 36122153

[22] Stival R. A. , Martins L. R. A. , Paganini J. , Caixeta G. N. , Manoel W. J. , de Paula E. C. , Ayres F. M. , Silva A. M. , Mühlbeier D. F. , and Saddi V. A. , Impacto do Fenótipo Triplo-Negativo no Prognóstico de Pacientes com Câncer de Mama de Uma Unidade de Referência no Brasil Central, Revista Brasileira de Mastologia. (2024) 22, no. 1, 6–12.

[23] Korde L. A. , Somerfield M. R. , Carey L. A. , Crews J. R. , Denduluri N. , Hwang E. S. , Khan S. A. , Loibl S. , Morris E. A. , Perez A. , and Regan M. M. , Neoadjuvant Chemotherapy, Endocrine Therapy, and Targeted Therapy for Breast Cancer: ASCO Guideline, Journal of Clinical Oncology. (2021) 39, no. 13, 1485–1505, 10.1200/JCO.20.03399. PMC8274745 33507815

[24] Harbeck N. , Penault-Llorca F. , Cortés J. , Gnant M. , Houssami N. , Poortmans P. , Ruddy K. , Tsang J. , and Cardoso F. , Breast Cancer, Nature Reviews Disease Primers. (2019) 5, no. 1, 10.1038/s41572-019-0111-2. 31548545

[25] Elston C. W. and Ellis I. O. , Pathological Prognostic Factors in Breast Cancer. I. The Value of Histological Grade in Breast Cancer: Experience From a Large Study With Long-Term Follow-Up, Histopathology. (1991) 19, no. 5, 403–410, 10.1111/j.1365-2559.1991.tb00229.x. 1757079

[26] Sparano J. A. , Gray R. J. , Makower D. J. , Pritchard K. I. , Albain K. S. , Hayes D. F. , Geyer C. E. , Dees E. C. , Goetz M. P. , Olson J. A. , Lively T. , Badve S. S. , Saphner T. J. , Wagner L. I. , Whelan T. J. , Ellis M. J. , Paik S. , Wood W. C. , Ravdin P. M. , Keane M. M. , Gomez Moreno H. L. , Reddy P. S. , Goggins T. F. , Mayer I. A. , Brufsky A. M. , Toppmeyer D. L. , Kaklamani V. G. , Berenberg J. L. , Abrams J. , and Sledge G. W. , Adjuvant Chemotherapy Guided by a 21-Gene Expression Assay in Breast Cancer, New England Journal of Medicine. (2018) 379, no. 2, 111–121, 10.1056/NEJMoa1804710, 29860917. PMC6172658 29860917

[27] Sung H. , Ferlay J. , Siegel R. L. , Laversanne M. , Soerjomataram I. , Jemal A. , and Bray F. , Global Cancer Statistics 2020: GLOBOCAN Estimates of Incidence and Mortality Worldwide for 36 Cancers in 185 Countries, CA-A Cancer Journal for Clinicians. (2021) 71, no. 3, 209–249, 10.3322/caac.21660. 33538338

[28] Shah R. , Disparities in Breast Cancer Care: A Global Perspective, Seminars in Oncology. (2021) 48, no. 4, 317–327.

[29] Li X. , Xu Y. , Luo Y. , Chen S. , Chen X. , Yang P. , and Zhang L. , Pathologic Complete Response (pCR) After Neoadjuvant Chemotherapy in Different Molecular Subtypes of Breast Cancer: A Systematic Review and Meta-Analysis, BMC Cancer. (2021) 21.

[30] Luen S. J. and de Azambuja E. , Neoadjuvant Systemic Therapy in Breast Cancer: Current Practice, Controversies, and Future Directions, Current Oncology Reports. (2022) 24, no. 10, 1347–1360.

[31] Wang P. , Wang X. , and Jiang Y. , Neoadjuvant Chemotherapy for Luminal Breast Cancer: A Comprehensive Review, Translational Oncology. (2021) 14, no. 10, 101183.

[32] Martínez-Sáez O. and Prat A. , Current and Future Management of HER2-Positive Metastatic Breast Cancer, JCO Oncology Practice. (2021) 17, 638–646, 10.1200/OP.21.00172. 34077236

[33] Ministério da Saúde (BR) , Instituto Nacional de Câncer José Alencar Gomes da Silva (INCA). Atlas da Mortalidade, 2021, INCA.

[34] Nicolini A. , Ferrari P. , and Duffy M. J. , Prognostic and Predictive Biomarkers in Breast Cancer: Past, Present and Future, Seminars in Cancer Biology. (2018) 52, 56–73, 10.1016/j.semcancer.2017.08.010. 28882552

[35] Ali B. , Mubarik F. , Zahid N. , and Sattar A. K. , Clinicopathologic Features Predictive of Distant Metastasis in Patients Diagnosed With Invasive Breast Cancer, JCO Global Oncology. (2020) 6, no. 6, 1346–1351, 10.1200/GO.20.00257, 32886558. PMC7529503 32886558

[36] Kim R. , Lim W. , Son A. , Kim S. Y. , Suh K. , Chang M. H. , Ryu J. M. , Kim S. W. , Nam S. J. , Lee J. E. , and Yu J. , Clinicopathological Features and Response to Neoadjuvant Treatment in HR+/HER2+ Versus HR−/HER2+ Breast Cancer, Cancers. (2022) 14, no. 18.

[37] Díaz-Casas S. E. , Castilla-Tarra J. A. , Pena-Torres E. , Orozco-Ospino M. , Mendoza-Diaz S. , Nuñez-Lemus M. , Garcia-Angulo O. , Garcia-Mora M. , Guzman-AbiSaab L. , Lehmann-Mosquera C. , Angel-Aristizabal J. , Duarte-Torres C. , and Vergel-Martinez J. C. , Pathological Response to Neoadjuvant Chemotherapy and the Molecular Classification of Locally Advanced Breast Cancer in a Latin American Cohort, Oncologist. (2019) 24, no. 12, e1360–e1370, 10.1634/theoncologist.2019-0300, 31346133. PMC6975950 31346133

